# Development and testing of a novel geothermal wall system

**DOI:** 10.1007/s40095-021-00407-y

**Published:** 2021-07-04

**Authors:** Matteo Baralis, Marco Barla

**Affiliations:** Department of Structural, Building and Geotechnical Engineering, Politecnico Di Torino, Corso Duca degli Abruzzi, 24, Torino, Italy

**Keywords:** Ground heat exchanger, Energy geostructures, Shallow geothermal energy system

## Abstract

Shallow geothermal energy systems have the potential to contribute to the decarbonization of heating and cooling demands of buildings. These systems typically present drawbacks as high initial investments and occupancy of wide areas. In this study, a novel energy wall system is proposed to overcome the limitations of conventional geothermal applications in urban areas. The system is characterized by ease of installation, low initial costs and applicability to existing buildings undergoing energy retrofitting. The paper illustrates the implementation of the prototype of such a system to an existing structure in Torino (Italy). An overview of the components is given together with the interpretation of an illustrative test carried out in heating mode. The data from both heating and cooling experimental campaigns allow us to highlight the potential of the proposed technology. The results suggest that an average thermal power of about 17 W per unit area can be exchanged with the ground in heating mode, while an average of 68 W per unit area is exchanged in cooling operations. The negligible impact on the stress–strain state of the wall and the surrounding soil thermal and hygrometric regime is also testified by the results collected. These aspects are associated with a reduced probability of interferences with other installations in highly urbanized areas, easiness of installation and affordable cost.

## Introduction

Decarbonization of heating and cooling systems for buildings plays a major role in the perspective of climate change mitigation. Air conditioning represents about half of the primary energy consumption in Europe [1]. In this respect, heat pumps have gained much attention in the last decades because they induce the electrification of the heating/cooling demands and the electricity needed can be easily produced from renewable sources [2].

Ground source heat pumps (GSHPs) are particularly suitable to this end because of the stability of the shallow geothermal energy with respect to weather and climatic conditions. GSHP systems are usually classified as closed-loop or open-loop schemes. While the latter generally allows for higher efficiencies, the former class of systems is safer from the environmental point of view since it does not exchange mass with the environment. Closed-loop schemes exchange heat with the subsoil employing a heat carrier fluid which is circulated by a hydraulic circuit. This medium is usually represented by a mixture of water and propylene glycol, namely a brine that allows to safely operate also at lower temperatures. The hydraulic circuit is in contact with the ground either directly or indirectly. Closed-loop schemes, however, require high initial investments due to excavation and drilling for circuit deployment. Based on the average power provided by a single BHE installation and on the installation, operation and maintenance costs, the price of thermal energy can raise to 80 €/kW. These costs can be reduced in the case of energy geostructures and very shallow geothermal systems, namely horizontal collectors or baskets. A detailed examination of the thermodynamic and hydraulic bases of the energy geostructures functioning is reported by Brandl [3]. A comprehensive overview of the different technologies of energy geostructures has been addressed by Adam and Markiewicz [4], while more recently a broad review of the topic was addressed by several authors [5, 6]. Initial costs are reduced for energy geostructures and very shallow geothermal systems by integrating the thermal exchanger with the structural elements, in the first case, or limiting the excavation needed in the latter case [7, 8]. Costs in these latter cases can be significantly reduced to about 50 €/kW (see Table 1).

**Table 1:** Comparison of advantages and disadvantages of the GeothermSkin system compared to direct market competitors. “ + ” symbols represent favourable aspects while “−” are related to problematic aspects

	Gas boiler	Horizontal collectors	Geothermal “Baskets”	Borehole Heat Exchangers	Energy Geostructures	GeothermSkin
Energy efficiency	+	−	±	+	±	±
Applicability to existing structures	+	+	+	+	−	+
Free land consumption	+	−	−	±	+	+
Initial costs	+	+	+	−	±	+
Running costs	−	+	+	+	+	+
Environmental costs	−	+	+	±	±	+
Cost per unit power [€/W]	~ 0.17	~ 0.5	~ 0.5	~ 0.8	N.A	~ 0.3

Virtually, all geotechnical structures can be equipped with heat exchangers to become energy geostructures. Most practical applications are related to energy piles as is shown by the relatively large amount of data collected by Di Donna et al.[9]. Energy piles were also the first applications to be studied in relation to the heating of buildings [10] and infrastructures [11] or in the field of de-icing technologies [12]. Because of the larger application, several authors studied in detail the thermal behavior of these structures [13] also coupling this aspect with the structural response [14] rather than with the profitability of the system [15]. More recently, growing attention was devoted to energy tunnels [16, 17] and energy walls [18] also in relation to de-icing applications [19]. In the case of energy tunnels, applications to the urban environment [20] and alpine settings [21] have been studied. The most common approaches involve experimental realization and numerical investigations both in the case of energy tunnels [22] and energy walls [23, 24]. Although energy geostructures have been successfully implemented [25, 26], this technology is mainly related to new constructions and has to be considered since the early stages of design [27].

Although very shallow geothermal systems can serve existing structures, they require the availability of large areas for installation that cannot be later occupied by buried pipes (e.g. sewers, district heating, gas distribution, etc.) neither by tall plants [28, 29]. This results in a strong limitation for densely inhabited areas where land scarcity influences urban planning policies.

This paper presents a novel very shallow energy wall system developed at the Politecnico di Torino to overcome some of the limitations affecting traditional energy geostructures and horizontal collectors. The system has been given the name of GeothermSkin, to highlight its characteristic to enhance the ‘underground skin’ of a building for heat exchange, and is in the process of patenting (Patent priority number: IT102019000024604). The system proposed can be applied to both new constructions and buildings undergoing refurbishment, using the earth-contact area of the retaining walls of the basements of the buildings. The system aims at being a robust solution in the field of shallow geothermal energy (SGE) [30] systems, merging the advantages of existing technologies in a single piece of equipment and overcoming some of the most problematic issues. A schematic comparison of the presented technology with other existing systems is given in Table 1. Particular attention was devoted to the comparison with similar geothermal systems. It should be noted that also in terms of costs, the absence of significant excavation works, both in terms of depths and areas involved, makes the GeothermSkin system a cost-efficient solution compared to other SGE systems.

The GeothermSkin system can be classified as an energy geostructure. Compared to traditional energy walls, the GeothermSkin system is conceived as a piece of external equipment to the earth-contact surface of the wall. For new constructions, the system can be implemented taking advantage of the open excavations during the first stages of the construction. In the case of existing buildings (e.g. for energy retrofitting), excavation work may be needed to expose the wall surface before installation.

The hydraulic circuit is made up of polymeric pipes (reticulated polyethylene, Pe-Xa, rather than high-density polyethylene, PE-D) that are simply fixed to the external surface once the wall construction is completed. Pipe deployment consists of straight sections and bends with 15 cm radii, equal to the maximum bending curve of the pipe employed. Pipes are temporarily ensured by clamps tiled into the wall acting as a support element. After pipes deployment, the excavation is backfilled and the ground ensures the long-term stable positioning of the pipes.

**Fig. 1:**
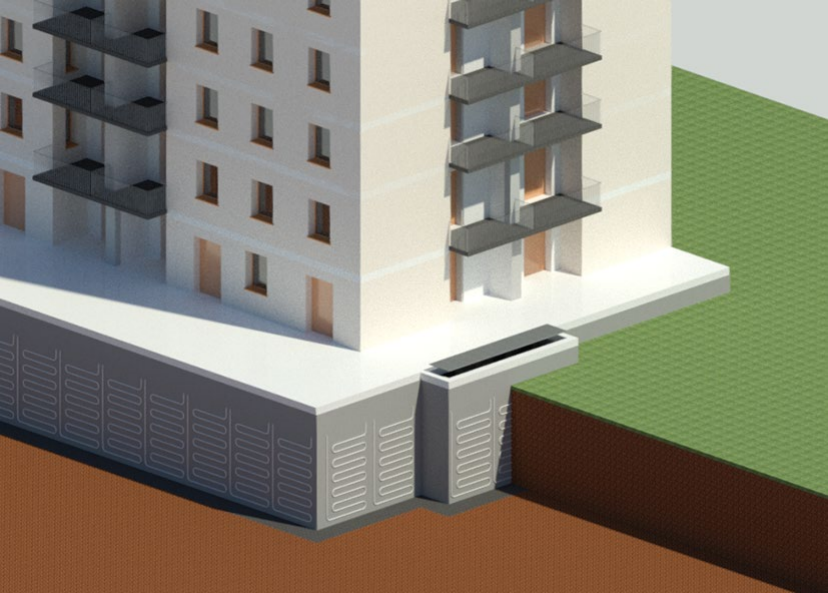
GeothermSkin energy wall heat exchanger conceptual application to a residential building

The system is conceived as a piece of modular equipment to ensure flexibility to the end-user, redundancy and the opportunity to exclude parts of the heat exchanger in the case of local damages or leakage, without compromising the entire system. This implies that the deployment is independent of the wall structural characteristics, differently from traditional energy geostructures whose circuit deployment is based on the reinforcement cage layout [27]. These features make the system optimal for deployment in existing buildings as part of retrofitting strategy. Also, for new constructions, the choice of such a solution can be made at any design stage, differently from classical energy geostructures that have to be included from the earliest phases of the design. Each module constitutes a hydraulic circuit where the pipes can be deployed preferentially in the horizontal rather than the vertical direction (see Fig. 1). The different deployments present peculiar benefits and drawbacks due to the path of the pipe. While the first deployment allows an easier elimination of air bubbles during circuit loading, the latter slightly diminishes the bends and hence the related hydraulic head losses. In both cases, the ends of each module are located at the opposite edges of the upper portion of the equipped area. This allows avoiding thermal short-circuiting among different branches of the circuit. Furthermore, the sequential linking of adjacent modules is made possible by such deployment in addition to the opportunity of parallel linking.

Although sequential linking allows limiting the flow rate needed at the heat pump while ensuring sufficient velocity of the heat carrier fluid in the ground heat exchangers, the thermal efficiency of parallel linking is expected to be higher.

In all cases, the heat exchanger pipes are connected to the main collector circuit on the internal side of the wall through appropriate holes. A manifold allows connection to the GSHP system.

The installation of the prototype of the GeothermSkin system took place in July 2019 at the Energy Center in Torino (Italy). The characteristics of the system and testing scheme will be described in the following sections. The results of the experimental campaigns in heating and cooling mode will be presented and described for an illustrative test, to demonstrate the potential of the technology.

## Materials and methods

**Fig. 2:**
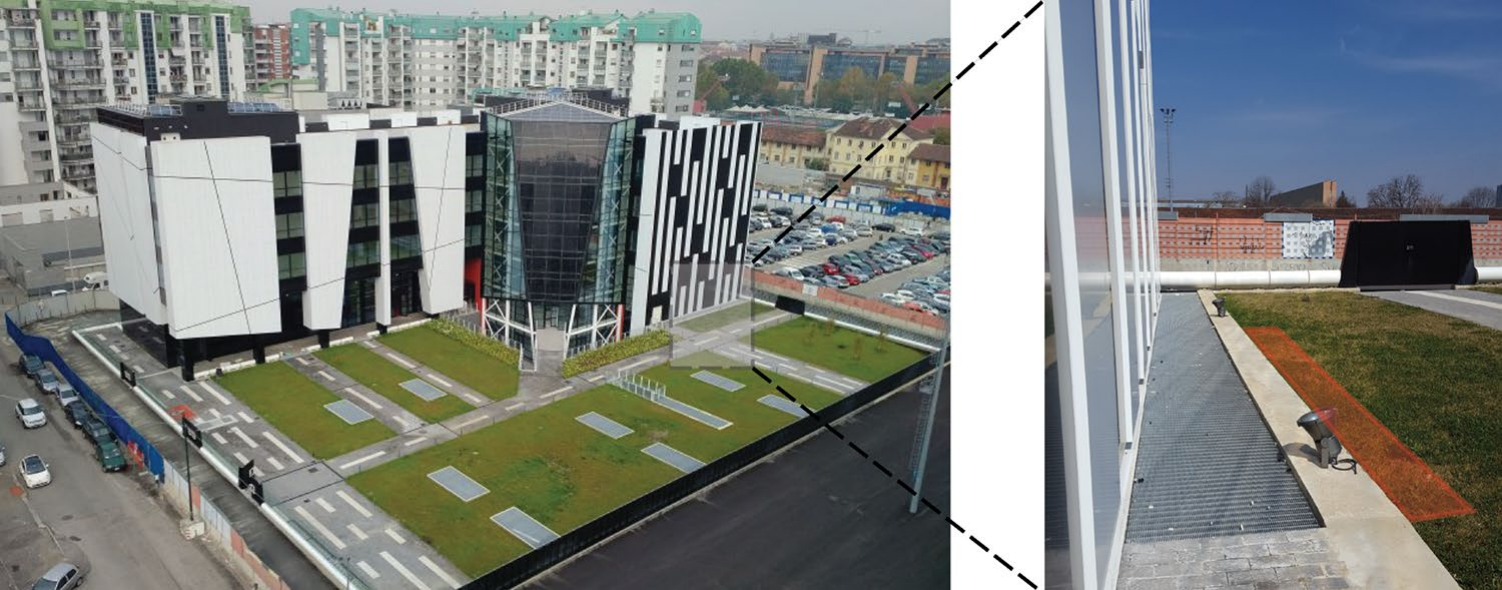
The Energy Center building with the location of the experimental site of the GeothermSkin system

To test the thermal performance of the newly developed GeothermSkin system, an experimental installation was built in July 2019 in the building hosting the Energy Center, an interdepartmental research aggregation in the Politecnico di Torino campus (Italy). Figure 2 shows the building and the location of the experimental site. The Energy Center hosts offices and research laboratories on a gross floor area of about 7000 m^2^. The building is characterized by high-efficiency standards and owns an energy-smart metering system to monitor the elevated and the underground storeys. The basement level covers a larger area with respect to the elevated building to host a large underground car park. The entire building together with its auditorium was operative before the installation of the GeothermSkin system. Because of this, the design of the experimental facility and the construction phases required new ground excavations and careful planning of interventions to ensure safety together with reduced times. The realization can be thus considered as an example of the application of the system to an existing building without affecting the serviceability of the internal activities. The existing structure where the system was installed is located at the north-eastern façade of the building. The wall faces on the outer side a green area with grass cover. On the inner side, the wall bounds an open air-technical corridor, namely a cavaedium, whose ceiling is represented by a walkable steel grid at the street level. This implies that the internal side of the wall is exposed to the temperature fluctuations of the external air. A relatively large distance from the underground car park serving the building ensures negligible influence on the thermal status of the green area facing the equipped wall.

### Geological setting

**Fig. 3:**
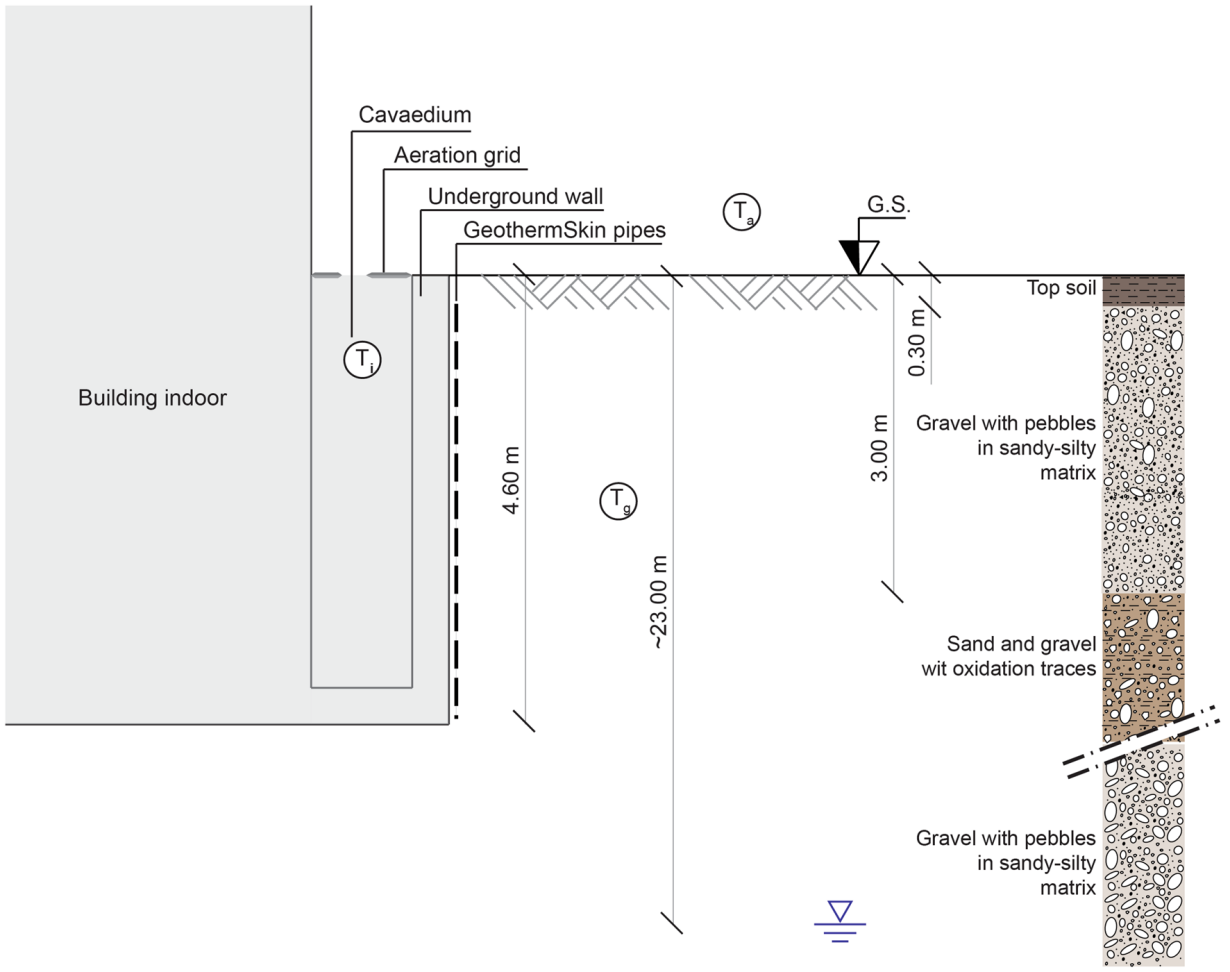
Cross-section of the location of installation of the GeothermSkin prototype with the indication of the stratigraphy

The metropolitan area of Torino lies on the end section of the Dora Riparia great alluvial plain. The upper 50 m of subsoil is mainly constituted by sand and gravel deposits with discontinuous and randomly distributed cemented layers from decimetric to metric width [31, 32]. These deposits show a wide variability with respect to density and cementation degree. This geological unit hosts an unconfined aquifer, characterized by a rather strong groundwater flow towards ESE, underlaid by low conductivity silty-clayey deposits. At the experimental site location, the water table is about 23 m below the ground surface [33], significantly below the depths interested by the prototype installation. As a consequence, no influence on the groundwater table neither on the groundwater temperature is expected from the activation of the system. The stratigraphy at the site is rather well known since several borehole samplings were carried out in relation to the geotechnical design of the buildings in the area [34, 35]. The results of sampling from a near borehole and the careful observation of the excavated material during the installation of the system allowed us to identify the stratigraphy shown in Fig. 3. Below 30 cm of topsoil, a 3-m layer of gravel with pebbles in a sandy and a locally silty matrix is present. This latter level was interested by the construction of the building. As a consequence, some inclusions of brick and concrete fragments were found within these depths. A sandy-gravelly deposit with clear evidence of oxidation and alteration interests lower depths.

### Site implementation

**Fig. 4:**
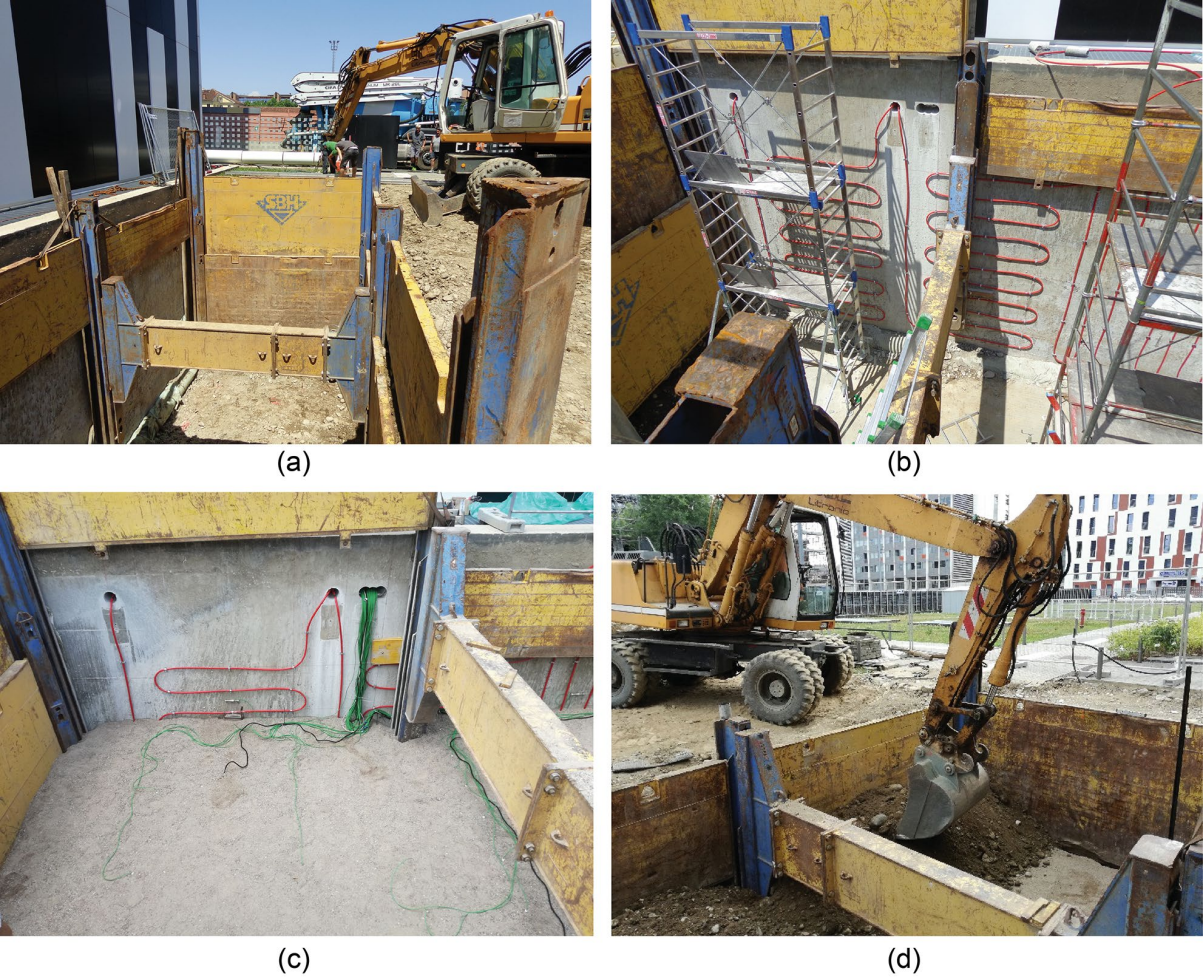
Installation phases of the GeothermSkin system prototype on an existing structure: **a** excavation, **b** pipe deployment, **c** monitoring sensors deployment and **d** backfilling

A portion of the Southern underground wall of the Energy Center (Fig. 2) was equipped with three modules of the GeothermSkin system. An excavation area was identified in the garden to bare the exterior of the retaining wall (see Fig. 4a). Once the excavation was completed and secured by appropriate iron shores, pipes were carefully fixed to the exterior wall surface using metallic clamps with an almost constant spacing of 0.75 m as shown in Fig. 4b.

**Fig. 5:**
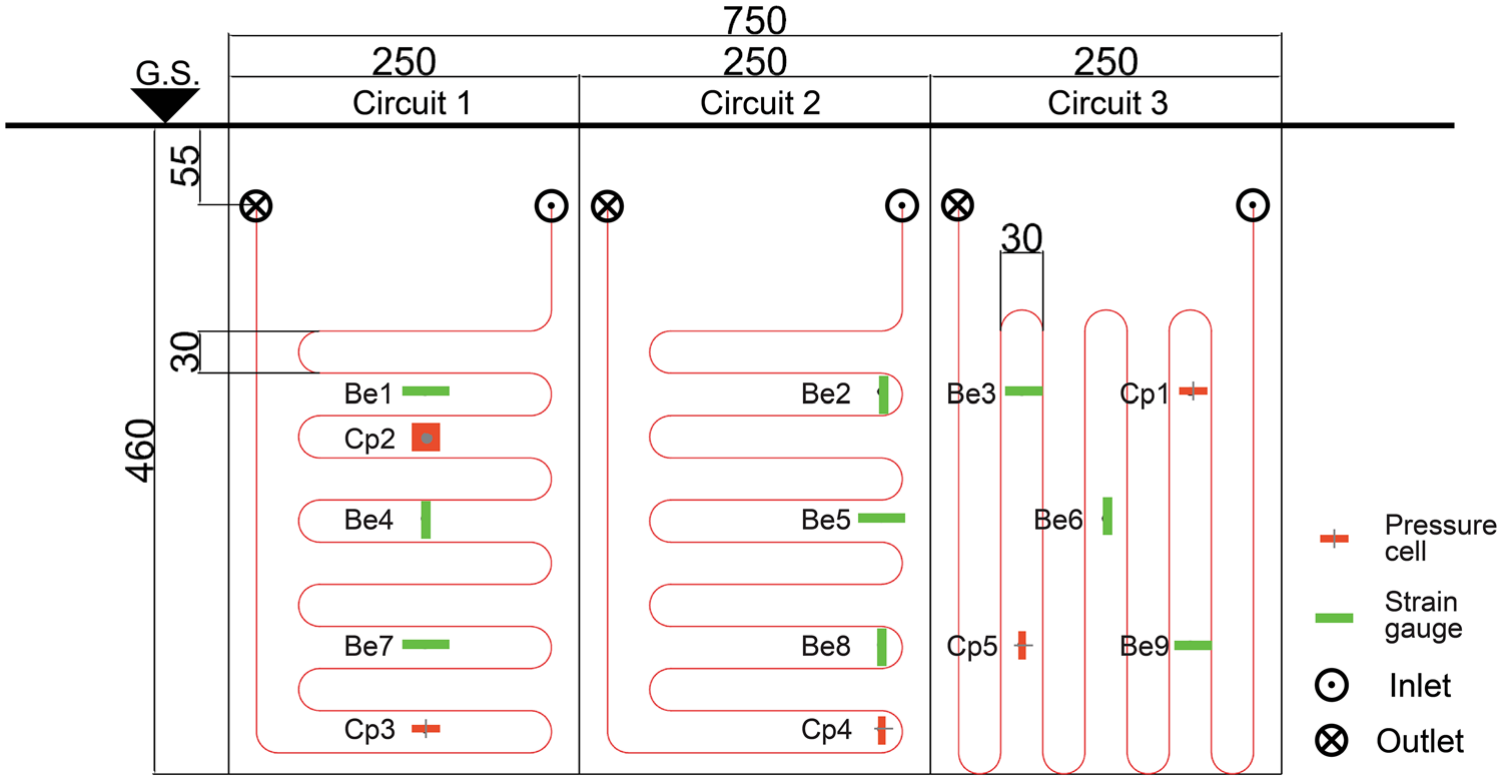
Experimental setup. Conceptual scheme of the heat exchanger circuits deployment along with the pressure cells (Cp) and the strain gauges (Be) for mechanical monitoring at the wall surface

Both pipe configurations were employed to allow comparative testing in identical site conditions. Two modules show a preferentially horizontal pipe deployment, while the remaining module shows prominently a vertical deployment (see Fig. 5). Both ends of each module are connected to the manifold placed at the inner side of the wall through dedicated drills in the wall structure. These holes were appropriately sealed afterward, to avoid seepage of water and durability issues on the wall. The above-mentioned manifold was properly designed to allow the connection of the modules in parallel or sequentially as well as the exclusion of each of them if needed. The experimental setup thus allows testing separately the thermal efficiency of every single module.

Collector and distributor DN32 PE100 pipes with heavy thermal insulation (20 cm thick closed elastomeric coating) connect the GeothermSkin circuits with the heat pump. On the contrary, the ground heat exchangers are constituted by highly conductive Pe-Xa pipes with a diameter of 20 mm. The thermal machinery is a reversible 3.15 kWt heat pump, commercially available on the market (model NIBE Fighter 1155-6). A fan coil of 1.5 kWt capacity simulates the user side, and the circuit is completed by a 100-l tank insulated by a thick insulation layer of polyurethane foam.

Once the hydraulic circuits were deployed, installation of the monitoring system and backfilling in multiple steps took place (see Fig. 4c, d).

### Monitoring and acquisition system

According to the experimental nature of the site, a comprehensive monitoring network was installed to observe the prototype performance both from a thermal and a structural point of view. Thermal activation may induce a variation of stresses and strains on the wall, as well as a variation of temperatures and saturation degree on the surrounding soil. The installed sensors are intended to give a comprehensive view of these aspects and are briefly described in the following order:

- Sensors on the wall to assess structural effects,
- Sensors embedded in the ground to assess environmental impact,
- Sensors on the hydraulic circuit to assess the energy performance.

#### Sensors on the wall

Local elongation and forces can be measured to assess the mechanical effects of thermal activation on the wall. Thus, a set of 9 resistive strain gauges and 4 oleo-dynamic pressure cells were firmly placed at the exterior surface of the wall using appropriate dowels in the desired position (see Fig. 5). The characteristics and location of the structural sensors are listed in Table 2.

**Table 2:** Stresses and strains sensors on the wall surface

Loop	Instrument	Depth (cm)	Axis	Code	Loop	Instrument	Depth (cm)	Axis	Code
1	Strain gauge	170	Horizontal	Be1	2	Strain gauge	350	Vertical	Be8
1	Pressure cell	200	Normal	Cp2	2	Pressure cell	410	Horizontal	Cp4
1	Strain gauge	260	Vertical	Be4	3	Strain gauge	170	Horizontal	Be3
1	Strain gauge	350	Horizontal	Be7	3	Pressure cell	170	Vertical	Cp1
1	Pressure cell	410	Vertical	Cp3	3	Strain gauge	260	Vertical	Be6
2	Strain gauge	170	Vertical	Be2	3	Pressure cell	350	Horizontal^a^	Cp5
2	Strain gauge	260	Horizontal	Be5	3	Strain gauge	350	Horizontal	Be9

^a^Sensor Cp5, originally designed with the horizontal axis accidentally rotated 15° in the counter-clockwise direction during backfilling

It should be noted that the axis orientation of strain gauges was carefully chosen on the circuits with horizontal deployment to obtain measures along two different directions at each of the depths monitored. Indeed the measurement axis corresponds to the sensor's main dimension.

On the contrary, pressure cells measure the local forces acting perpendicularly to the 200 × 200 mm plate that is internally filled by oil. The oil is indeed pressurized within the plate so that the internal pressure of the fluid continuously balances the external pressure. Accurate calibration of the signal was carried out before installation. To reconstruct mechanical effects in the three dimensions, measurement plates were deployed to monitor stresses not only in the horizontal and vertical direction but also perpendicularly to the wall surface. All the mechanical sensors are also equipped with a PT-100 sensor for temperature measurement. Signals from sensors are converted based on the calibration operated before installation. This allowed taking into account the different lengths of the cables from the instrumented point to the acquisition unit. Measurement range, accuracy and field of observation are listed in Table 3.

**Table 3:** Features of the sensors included in the monitoring system

	Strain gauge	Pressure cell	PT-100	Hygrometer	Tensiometer	Energy meter
Measurement range	−1500/ + 1500 με	0/6 MPa	−40/ + 60 °C	0/80%	−0.009/100 kPa	0.5/3 m^3^/h −15/ + 50 °C
Resolution	1 με	0.1 MPa	0.01 °C	10^–4^	0.1 kPa	10 W
Accuracy	0.2%	0.3%	0.1 °C	0.03%	10% + 2 kPa	50 W
Working conditions	−10/ + 50 °C	−20/ + 60 °C	−40/ + 60 °C	−20/ + 60 °C	−40/ + 60 °C	–

#### Sensors in the ground

**Fig. 6:**
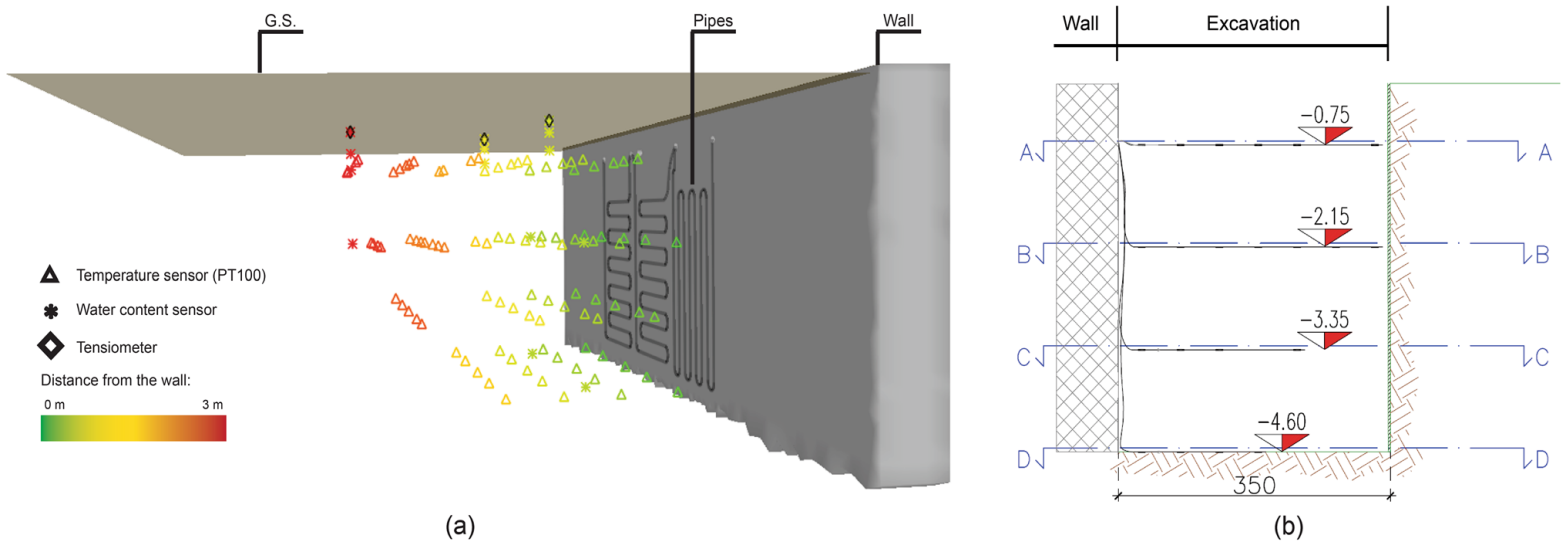
Ground monitoring system. **a** 3D scheme of the sensor location; **b** transverse cross-section

The second group of sensors is constituted by probes embedded in the ground. The backfilling was carried out in multiple steps to allow the installation of 96 PT-100 temperature measurement points, 18 water content sensors and 3 tensiometers (Fig. 6a). Indeed, a large number of temperature sensors were placed at four different depths: 0.75 m, 2.15 m, 3.35 m and 4.60 m below the ground surface. These instrumented levels were identified with a progressive letter: A, B, C and D. Each layer was compacted and leveled before placing the probes. PT-100 were grouped in thermometric strings of 4–8 points whose alignment results staggered on the three dimensions (see Fig. 6). Water content sensors were placed at 5, 30 and 60 cm depth along three different vertical alignments to allow for detecting the high gradients expected at the surface as a consequence of rainfall infiltration. Suction potential measurements are operated by tensiometers with an inner ceramic porous matrix. In order to allow for precise measurement, the ceramic matrix was saturated before burial. To this end, the tensiometer was immersed for about 15 h before installation. Environmental sensors characteristics are listed in Table 3.

Sensors’ cables were carefully deployed to accommodate the expected ground settlements after backfilling. The acquisition unit was placed near the distribution manifold and is equipped with a router module for direct upload of the data via GSM to a dedicated cloud portal. All parameters are recorded continuously, and the acquisition frequency can be adjusted remotely.

#### Sensors for energy performance assessment

The third group of sensors is devoted to the measurement of circuit variables. Energy performance is assessed by the use of three energy meters, installed close to the ground heat exchangers. The deployment allows to separately account the performances of each module as well as of the whole system.

The operative parameters of the heat pump are also recorded continuously by using the USB port. Acquisition frequency can be adjusted through the heat pump control unit. Functioning can be monitored remotely from a dedicated web portal thanks to an Ethernet connection. Monitored parameters include also the air temperature in the cavaedium (T_i﻿_ in Fig. 3) through a dedicated PT-100 sensor.

Climate data (external air temperature, T_a_, rainfall, relative humidity, solar radiation and wind speed) are measured from a weather station located at about 400 m from the experimental site. Weather data are recorded every 15 min.

### Testing program

The installation of the system was completed in July 2019. Circuit saturation with a 25% in volume mixture of water and propylene glycol and preliminary testing against leakages were carried out till September 2019. This period also allowed to reach the thermal equilibrium in the ground and complete consolidation, after the alterations induced by the excavation and backfilling.

The experimental setup allows testing the system's performances and its effects in multiple conditions. Thanks to the reversibility of the heat pump, both heating and cooling modes can be simulated.

A manifold governs the activation and the linking of the different ground heat exchanger modules. Hence, several configurations can be tested separately, depending on the way the valves are controlled:

- Sequential linking of the three circuits;
- Sequential linking of circuits 1 and 2 rather than circuits 2 and 3;
- Parallel linking of the three circuits;
- Parallel linking of two circuits;
- Single circuit.

An experimental campaign to test winter and summer performances started in late September 2019. The main focus was to assess the efficiency of different deployments of the GeothermSkin modules; therefore, most tests were performed with the same heat pump settings. Only in two cases during the heating season, the target temperature to be delivered to the user was changed to 35 and 55 °C.

First tests were performed in cooling mode and then changed into a heating mode during winter 2019/2020, before interruption due to the SARS-CoV-2 outbreak. The experimental campaign was later recovered in spring 2020 with new cooling mode tests. The full list of the experiments and their characteristics is given in Table 4. A total of 18 tests were performed, out of which 11 in heating and 7 in cooling mode. At the end of each test, the system was turned off for long enough to guarantee that the ground could recover thermal equilibrium as testified by the monitoring system.

**Table 4:** Main features of the experimental tests carried out

Test	Start date	End date	Mode	Target temp (°C)	Active modules	Link	Duration [h]	Flow rate [l/h]
C1	16/09/2019 10:30	19/09/2019 10:36	Cooling	5	2; 3	Sequential	72.1	575
C2	23/09/2019 10:50	25/09/2019 11:00	Cooling	5	3	-	48.2	575
H1	24/10/2019 11:20	20/11/2019 14:20	Heating	45	1; 2; 3	Sequential	651.0	670
H2	28/11/2019 12:25	02/12/2019 16:00	Heating	45	1; 2	Sequential	99.6	546
H3	07/12/2019 10:00	10/12/2019 09:25	Heating	45	2; 3	Sequential	71.4	530
H4	20/12/2019 19:30	13/01/2020 10:53	Heating	45	1; 2	Parallel	567.4	910
H5	24/01/2020 17:27	28/01/2020 09:20	Heating	55	1; 2	Parallel	87.9	910
H6	31/01/2020 17:56	03/02/2020 09:26	Heating	35	1; 2	Parallel	63.5	923
H7	14/02/2020 19:20	17/02/2020 09:20	Heating	45	2; 3	Parallel	61.8	926
H8	21/02/2020 18:04	24/02/2020 10:04	Heating	45	1; 3	Parallel	62.0	282
H9	28/02/2020 18:40	02/03/2020 09:25	Heating	45	1; 3	Parallel	64.0	931
H10	05/03/2020 19:25	07/05/2020 10:00	Heating	45	1; 2; 3	Parallel	62.8	1006
H11	01/06/2020 10:37	05/06/2020 12:50	Heating	45	1	-	1502.6	713
C3	16/06/2020 11:48	26/06/2020 09:48	Cooling	5	1; 2	Parallel	98.2	674
C4	10/07/2020 16:05	21/07/2020 12:40	Cooling	5	1; 2	Sequential	238.0	394
C5	07/08/2020 12:25	03/09/2020 12:48	Cooling	5	2; 3	Parallel	260.6	682
C6	15/09/2020 11:12	24/09/2020 09:12	Cooling	5	1; 2; 3	Parallel	648.4	731
C7	14/02/2020 19:20	17/02/2020 09:20	Cooling	5	2; 3	Sequential	214.0	392

## Results and discussion

Throughout all the tests, the temperature changes at the primary circuit end were monitored to assess the performances of the system. The thermal exchange was computed accordingly to the equation below:

1 $$q = m'c\left( {T_{{ret}} - T_{{lin}} } \right)$$

where *T*_ret_ and *T*_lin_ are the return and the line temperature of the fluid, m’ is the mass flow rate (given by the volumetric flow rate and the specific weight product), and c is the specific heat capacity, according to the heat carrier fluid brine that filled the hydraulic circuit. While the volumetric ratio of water over propylene glycol is given, thermal and physical parameters of the fluid slightly depend on the fluid temperature, as reported in Table 5.

**Table 5:** Heat carrier fluid properties within the temperature range typical of GeothermSkin functioning

Propylene glycol to water mixture		(%)	10	20	30	40	50
Temperature, *T*		(°C)	25	25	25	25	25
Specific weight, *ρ*		(kg/m^3^)	1027.55	1023.44	1018.85	1013.79	1008.28
Specific heat capacity, *c*		(KJ/kgK)	3.893	3.917	3.941	3.965	3.988
Thermal conductivity		(W/mK)	0.456	0.467	0.478	0.488	0.496

The mass flow rate is specific to the test interpreted because of the variation of the mean flow rate throughout the tests (Table 4). Variability of flow rates is partly due to the hydraulic head losses that in turn depend on the length and the deployment of the modules tested. However, prevailing influence is exerted by the circulation pump settings and the time the compressor was active. This time is strictly related to the temperature of the fluid delivered to the user.

Integration of Eq. (1) allowed to define the mean thermal power exchanged during the test in addition to the peak thermal power and the exchange rate per unit area of the equipped surface (Table 6).

**Table 6:** Experimental test results

Test	Mean inlet temperature (°C)	Mean ground temperature (°C)	Peak thermal power (kW)	Mean thermal power (W)	Mean exchange rate (W/m^2^)
C1	39.7	25.8	13.7	1695	73.7
C2	24.1	25.6	10.6	554	48.2
H1	11.7	20.2	3.8	615	17.8
H2	8.2	15.3	2.4	414	18.0
H3	7.2	13.8	1.9	411	17.9
H4	6.9	11.8	2.1	492	21.4
H5	5.5	9.6	2.3	548	23.8
H6	8.2	9.3	2.6	333	14.5
H7	6.9	9.9	2.3	478	20.8
H8	12.2	10.3	1.8	115	5.0
H9	8.8	10.7	2.5	445	19.4
H10	8.3	10.9	2.5	476	13.8
H11	11.9	13.0	3.3	363	31.6
C3	32.8	18.8	5.9	1615	70.2
C4	35.4	20.1	6.0	1640	71.3
C5	39.3	23.3	10.1	1766	76.8
C6	38.0	25.7	8.9	1821	52.8
C7	39.8	25.8	7.4	1904	82.8

^a^The ground temperature is assumed as the temperature at mid-depth and at distance from the wall (reference to sensor C8T7)

**Fig. 7:**
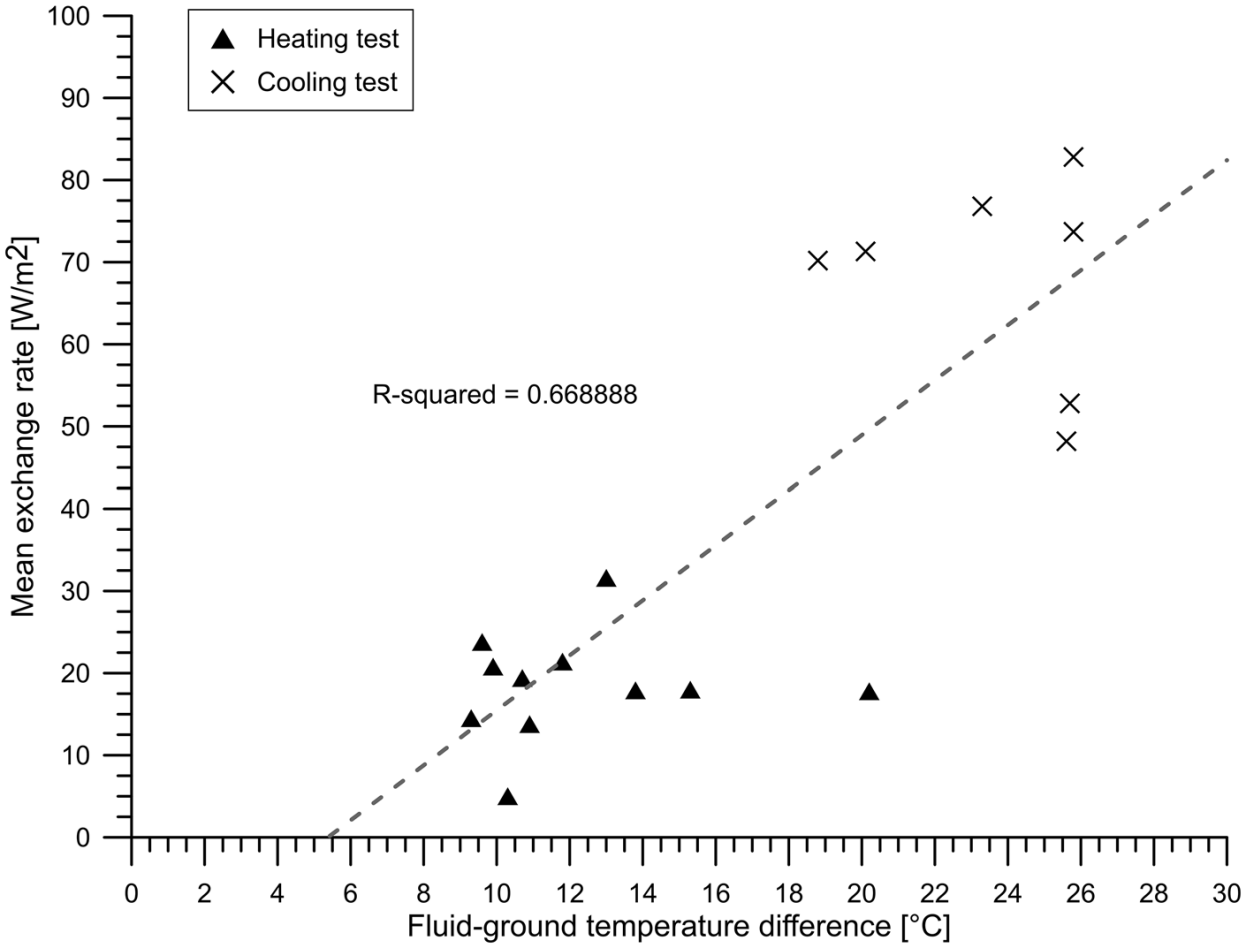
Difference in fluid and ground temperature against GeothermSkin heat exchange rates obtained experimentally

Extremely high efficiency of the system was shown in cooling operation. The mean exchange rate of the GeothermSkin module was found to be 18.6 W/m^2^ and 68.0 W/m^2^ in heating and cooling mode, respectively. Although the heat flux towards the inner side of the wall was not measured, it is believed that most of the heat flux is exchanged with the ground. The positive relationship of the mean exchange rate with the mean temperature difference between the fluid and the ground corroborates this assumption. Although a non-negligible dispersion around the trend is shown in Fig. 7, a clear distinction between tests in heating rather than in cooling mode is obtained and confirms that higher temperature differences lead to higher heat exchange rates.

This dispersion can also be interpreted in the light of the different circulation of the heat carrier fluid related to the modules activated in each of the tests. Furthermore, also the target temperature to be delivered to the user and the heat carrier fluid velocity exert an influence on the heat exchange rate.

**Fig. 8:**
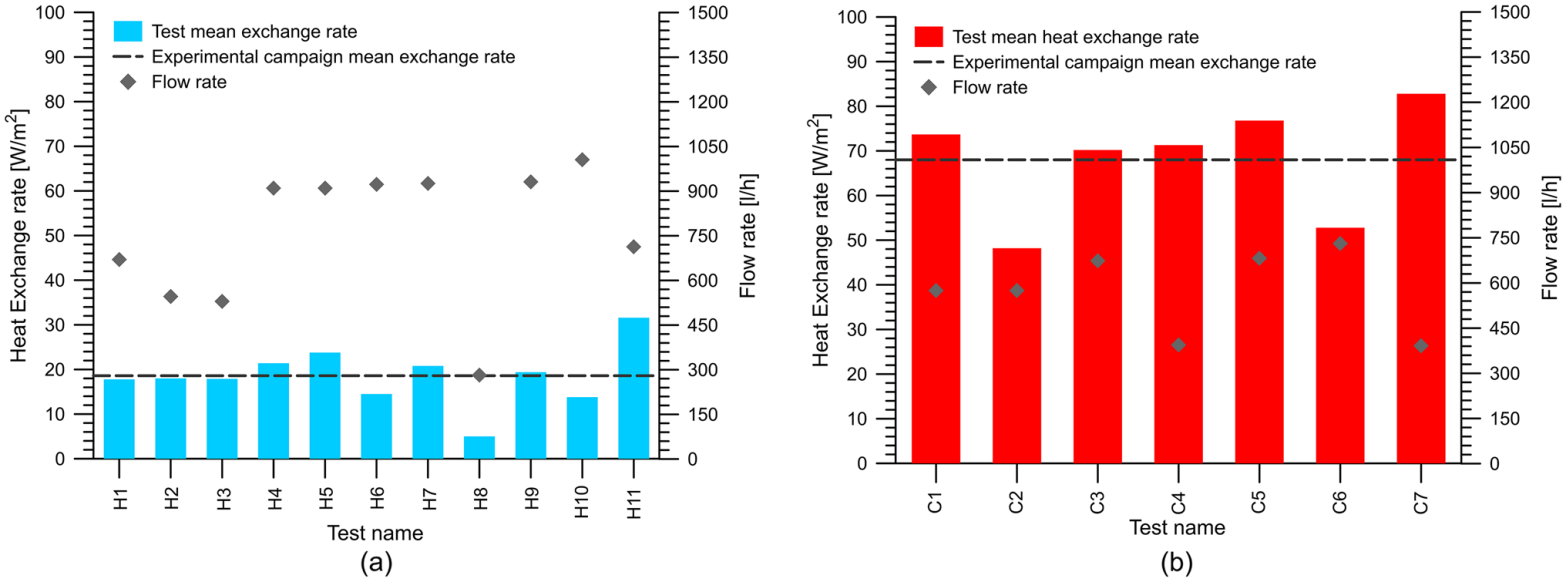
Experimental test results from **a** heating mode and **b** cooling mode campaigns

Figure 8 shows the heat exchange rate obtained for each test carried out together with the fluid flow rate.

It should be noted that in most cases during the heating campaign the mean exchange rate was particularly stable within the range of 15–25 W/m^2^. Only in the case of test H8, a particularly low value of the heat exchange rate was obtained. This is likely related to the particularly low value of the flow rate. Although no clear relationship of flow rate with heat exchange rates appears, it is believed that particularly low flow rates result in inefficient heat exchange due to the limited temperature difference that can be obtained with the ground.

Another aspect to take into account when dealing with thermal efficiency is the length of the exchanging circuit. It should be noted that in the heating mode the most efficient configuration is represented by the functioning of a single module. On the contrary, the same configuration leads to the least efficient performance. This might be related to the high-temperature gradients that characterize the cooling mode and that are not completely exhausted in the length of the circuit of a single module. Indeed the difference of the temperature at the outlet side of the circuit in test C2 with the ground is still large. This difference can be reduced by adding another module, linked sequentially to the first one (e.g. test C1) leading to higher thermal performances.

The additional path represented by the sequential link with another module seems to increase the overall efficiency of the heat exchange. On the contrary, the additional area to be equipped in sequential links is not justified in the case of heating where the temperature difference of the fluid with the ground at the outlet end of the first module is sufficiently small. This hence results in lower heat exchange rate values in the connection of further modules.

The comparison of the results in terms of the exchange rate, particularly between test H2 and H3, denotes that the thermal efficiency is virtually equal for circuits with the horizontal preferential direction of pipes and the ones with the preferentially vertical direction. Thus, the choice of the best solution has to be based exclusively on the basis of hydraulic considerations.

In this regard, measures of the flow rate against circulation pump head demonstrated that hydraulic head losses are virtually equal for both horizontal and vertical deployment. This evidence further confirms that from a geometry point of view at equal conditions of installation, the length of the pipes is a key aspect to consider. As a consequence, it is believed that in most conditions, the best solution is the horizontal pipes deployment. The shape of the circuit decreases the issues related to circuit charging and allows easier venting of air bubbles.

**Fig. 9:**
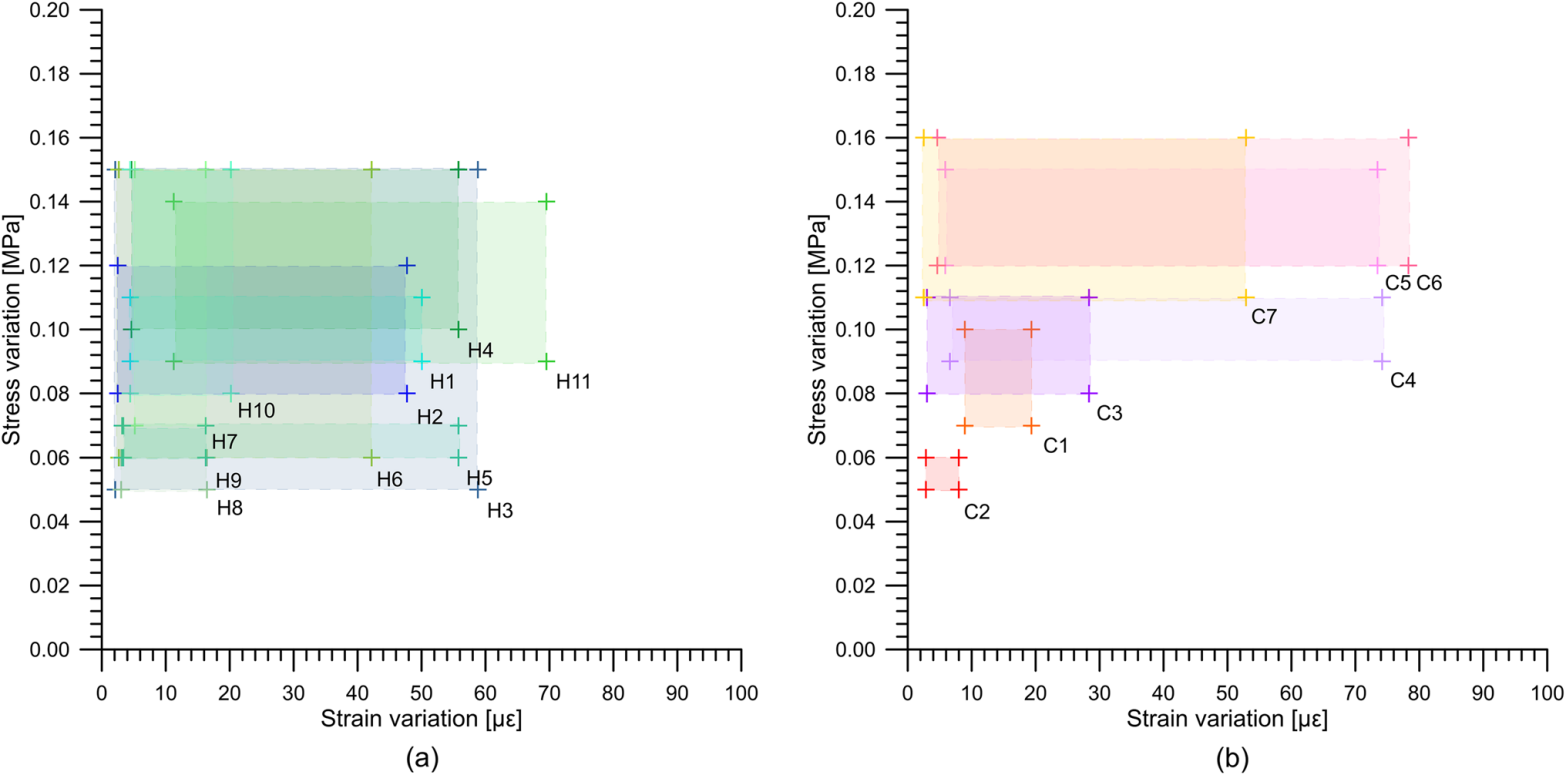
Stresses and strains variations experienced during the tests in **a** heating mode and **b** cooling mode. For each test, the ID is indicated at the lower right corner of the variation field

The wide monitoring network allowed us to analyze the effects of the GeothermSkin modules on the structural integrity of the supporting wall. In Fig. 9, the variations of the mechanical variables stresses and strain in different locations and directions, measured during the experimental campaigns, are reported. The changes experienced due to the thermal activation are relatively small. The low-temperature variations at the wall/ground interface result in extremely small changes (lower than 0.15 MPa) in the stresses measured through the pressure cells. It should be noted that the pressure cell measurements could be partly underestimated due to slight accommodation within the ground after backfilling; nevertheless, they are considered reliable based on the comparison and good agreement to the strain variations.

Virtually equal stress variations were experienced during the summer cooling season (Fig. 9b), while the strains were limited to about 80 με. This value is slightly higher than the values obtained in the winter cooling season when the value of 60 με was exceeded only once. The collected data confirm that the additional actions that are exerted on the underground structure due to the thermal activation of the GeothermSkin modules are negligible from the practical point of view. This is partly related to the external deployment that allows also to partially accommodate the thermally induced strains of the pipes and to further limit the thermal variations within the structural parts of the wall.

Results thus suggest that application of the GeothermSkin system to underground surfaces can be done without any consequence on the serviceability of the supporting walls as well do not require any variation on their structural design. It follows that application to existing or already designed structures does not request specific adjustments.

### Illustrative test interpretation

An illustrative test in heating mode is analyzed in detail in this section. All tests listed in Table 4 were interpreted accordingly to the methodology described in the following.

The test H2, which is considered here, involved activation of the circuits with preferential horizontal pipes deployment (modules 1 and 2 in Fig. 5), with the sequential connection. Test main features are listed in Table 7. The temperature desired at the user side, namely the climatic curve, was chosen to be independent of the external air temperature and equal to 45 °C to ensure continuous functioning during the approximately 4-day duration of the test.

**Table 7:** Illustrative test main features

Test start time	(gg/mm/aa hh:mm)	28/11/2019 12:25
Test end time	(gg/mm/aa hh:mm)	02/12/2019 16:00
Duration, *d*	(h)	99,6
Operative mode	(–)	Heating
Active circuits	(–)	1–2
Link	(–)	Sequential
Target user temperature, *T*_*T*_	(°C)	45
Ground side flow rate, *q*	(l/h)	546
Test code	(–)	H2

**Fig. 10:**
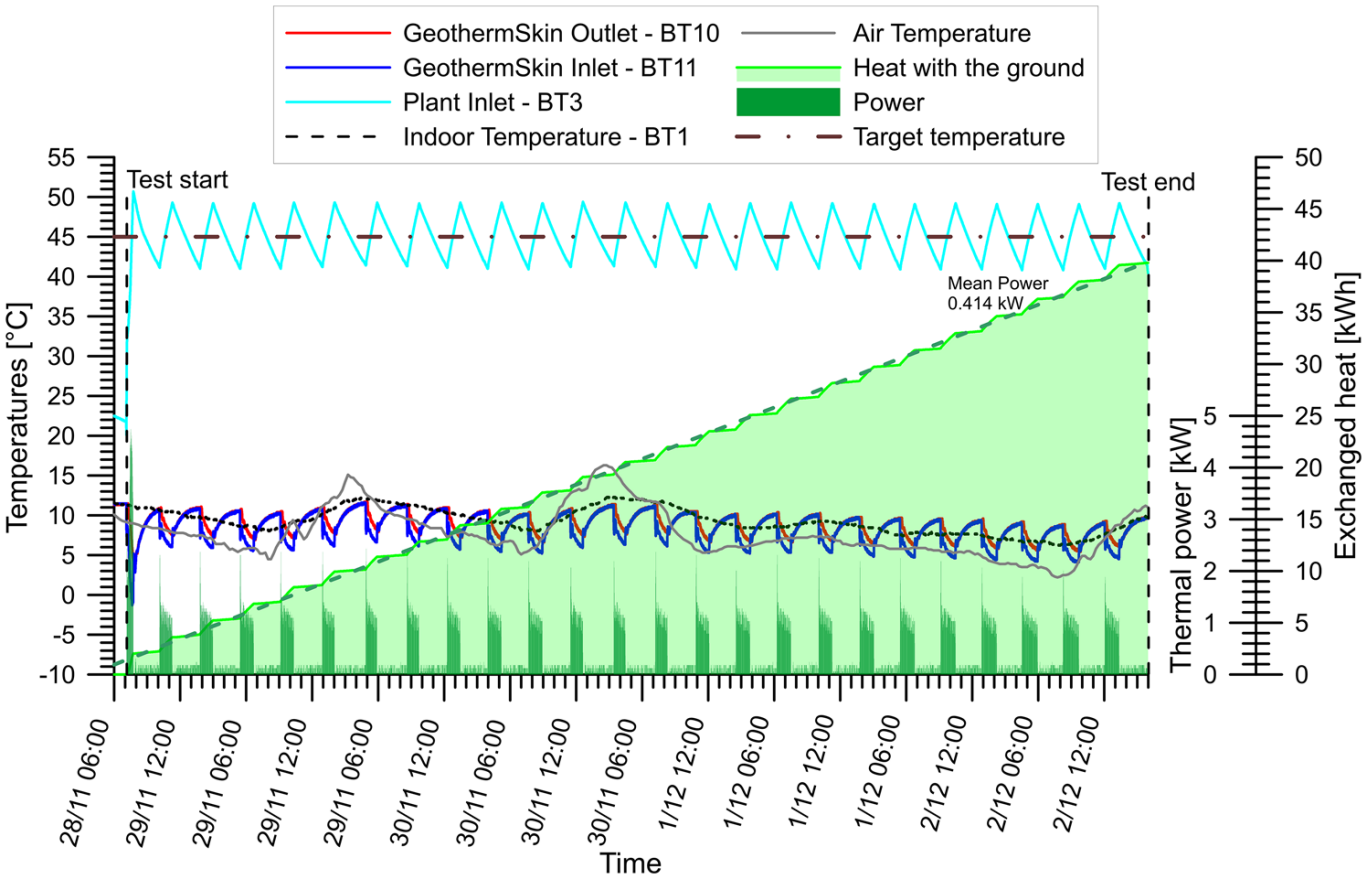
Monitored temperatures in the circuit and at the experimental site during test H2

Temperatures recorded at the primary circuit end are reported in Fig. 10 along with the instantaneous thermal power exchanged and the cumulative heat provided by the ground. It can be appreciated that the temperature at the user side, represented by the light blue continuous line, oscillates cyclically around the target temperature of 45 °C (line-dot line). Apart from the initial ramp that drives the heat carrier fluid in the puffer tank from the ambient temperature to the target one, the GeothermSkin inlet and outlet temperatures show the superposition of two trends: a short-term variation and a baseline shift.

The short-term cyclic variation is associated with the heat pump compressor cycles. Compressor cycles are in turn composed of two phases, active and passive, respectively.

In the active phase, the heat pump raises the heat carrier fluid temperature at the user side until the return temperature is in line with the target value. In this phase, electricity is used by the compressor and the hydraulic circulation pumps. Once the desired temperature is obtained, the compressor stops and the passive phase starts. Here, the electricity is used only for fluid circulation. In test H2, the compressor cycle lasted approximately 220 min out of which 70 min represents the active phase. During the active phase, the temperature at the GeothermSkin inlet decreases by 5.0 °C while the outlet temperature drop is just 3.3 °C.

The alternation of active and passive phases is also testified by the correspondence of the active phases with the peaks of the exchanged thermal power shown in Fig. 10. Although the exchanged heat shows an almost regular rise during the whole duration of the test, the green line showing the cumulative energy exchanged shows regular steps. The interpolation of the cumulative energy from the beginning to the end of the test testifies an almost constant thermal efficiency with a mean exchanged power of 414 W. A baseline shift superimposes the cyclic effect of the compressor activity on the temperatures recorded in the GeothermSkin system (see Fig. 10). This baseline shift represents the smooth influence on the circuit thermal status of the temperature inside the cavaedium (dotted line in Fig. 10). This is in line with the relevance of the wall thermal boundary condition, according to previous literature [18]. The indoor temperature trend of Fig. 10, compared to the trend of the air temperature, shows significant damping of the daily thermal variations. Also, a small phase shift can be observed. It should be noted that the temperature within the heat exchanger circuit is regularly higher than the air temperature. This testifies the higher thermal stability of the ground and, theoretically, higher efficiency with respect to an air source heat pump.

The temperature difference between GeothermSkin inlet and outlet reaches a considerable value of 3.9 °C corresponding to a peak power of 2.37 kW_t_. It should be noted that during the passive phase of the compressor cycles, the temperature drop is completely recovered. This is highlighted by the typical ascending asymptotic trends of fluid temperatures in Fig. 10. This is also due to the continuous fluid circulation during the whole test duration. The high thermal capacity of the ground volume involved by heat exchange is testified by the slower response of the inlet temperature with respect to outlet one. This results in the high mean exchanged power that corresponds to an average power of 18 W per equipped unit area (see Table 6).

These findings are in line with the thermal efficiency of classic energy wall systems [9, 36] and slightly above documented cases in similar climatic conditions [37].

**Fig. 11:**
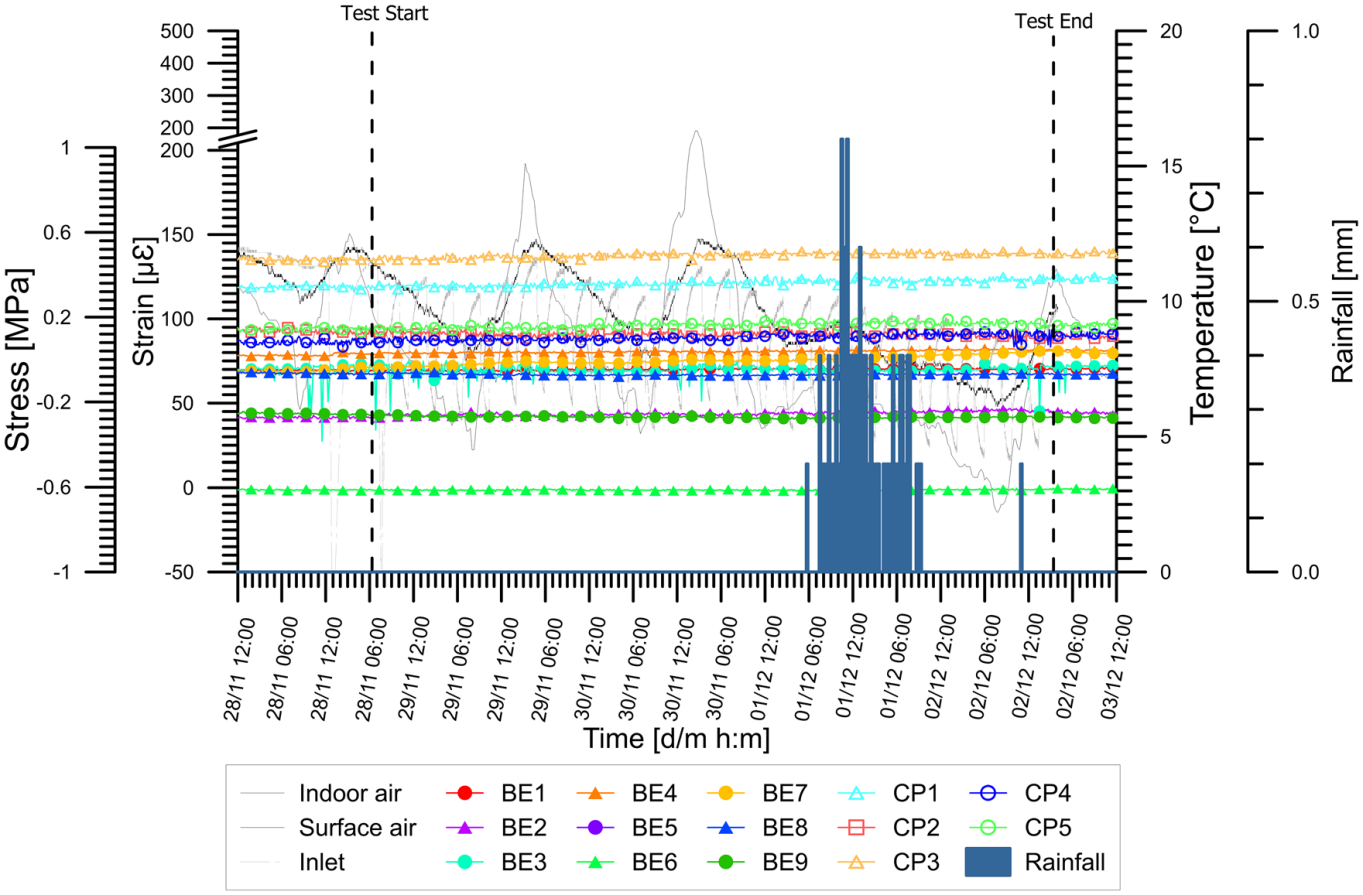
Monitored structural effects on the external wall surface during test H2

A negligible variation of the stresses (less than 0.1 MPa) and strains (in the range 5–40 με) fields measured on the wall is documented in Fig. 11. This is in agreement with the observations derived from the full experimental campaign previously shown in Fig. 9 and is consistent with the small difference between the indoor air and the heat carrier fluid temperatures (Fig. 10).

**Fig. 12:**
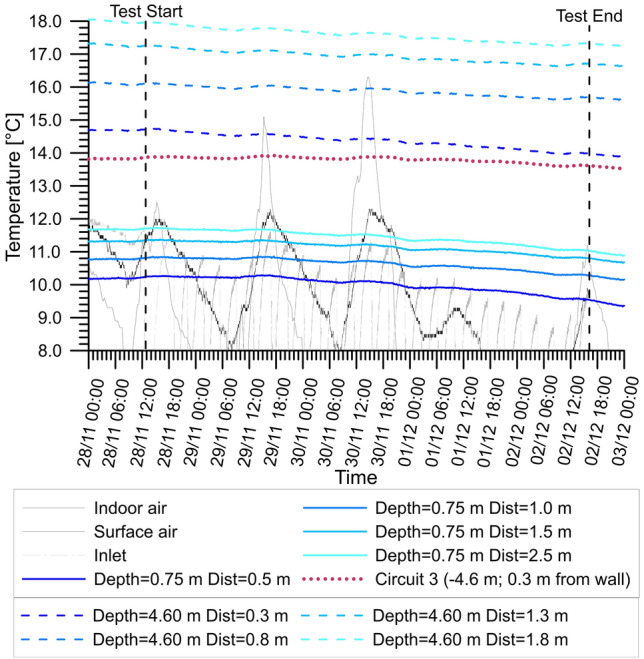
Monitored temperatures within the ground during test H2

The thermal variations within the ground are also very limited and are shown in Fig. 12. The influence exerted on the ground temperatures by the cool boundaries (the ground surface and the indoor air) can be inferred by comparing the trends registered by sensors placed at different depths (e.g. 0.75 m and 4.60 m depth, respectively). It appears that the temperatures at the deeper level are slightly higher, in line with expectations during the winter season [38] where the deeper strata are less influenced by the ground surface. Furthermore, the temperature difference measured is higher close to the wall. This is shown clearly in Fig. 12 where the dotted lines are shown to be more spaced than the continuous lines. All the sensors aligned to the activated circuits show a decreasing trend. On the contrary the red dotted line, showing the temperature in front of the non-activated GeothermSkin module, is more stable. The temperature decrement in front of the activated modules is more noticeable next to the wall rather than at a certain distance. Although this effect might be due to the cooler internal air, most of this influence can be assumed from the thermal activation due to the direct contact with the ground and the slightly lower temperature values with respect to the temperature registered in the cavaedium. As a consequence of the above observations, it can be inferred that the thermal influence exerted by the thermal activation is limited to a restricted area in the proximity of the wall.

The results from the experimental campaign suggest that the thermal affection of the ground is rather limited in extension and magnitude. This would result in a remote risk of causing interferences with other shallow geothermal energy systems as the thermal energy is preserved at a short distance from the wall. Also, the risk of overexploitation of the subsurface resource is expected to be extremely low.

## Conclusions

Shallow geothermal energy has been proved to be an effective solution in decarbonization of the energy demand of buildings despite the high initial investment costs and the land scarcity issues in urban areas. A new energy wall system that broadens the application of the energy geostructures concepts to existing buildings, retrofitting and new structures is here proposed.

The GeothermSkin energy wall system allows the external equipping of the earth-contact area of buildings with a modular ground heat exchanger with virtually no horizontal area occupancy. The modularity of the system allows for higher robustness because of the ability to isolate damaged portions during construction or operation. This results in reduced risks of incomplete satisfaction of expected thermal power outputs.

The proposed system, in its two distinct configurations, was proved to be a cost-effective solution by the construction of a prototype in an existing building. Preliminary assessment of cost per unit power generated shows values of about 30 €/kW, mainly related to labor costs during installation. Nonetheless, further investigation should be carried out considering full-scale applications and comparison to the energy demand of existing buildings. Furthermore, standardization of the construction process may even improve cost–benefit analysis.

A prototype installation of the GeothermSkin system allowed the preliminary assessment of the thermal performances in the value of 16.8 W/m^2^ in winter operation and in the range of 48.2–82.8 W/m^2^ in summer conditions with the particularly promising mean value of 68.0 W/m^2^. The thermal performances obtained experimentally confirm the ability of such system of keeping stable the efficiency as a consequence of the persistence of a high temperature difference of the ground with the heat carrier fluid and a consequent high thermal flux.

The wide monitoring network installed at the experimental facility allowed to determine that no relevant affection of the stresses and strains fields on the wall surface is experienced during operations due to the small variation of temperatures on the ground surface with respect to natural cavaedium temperature oscillations. Also the thermal status of the ground was proved to be interested by a small-dimension plume, thus limiting the risk of thermal interferences even in highly urbanized areas.

## Data Availability

The data generated during the current experimental campaigns are available from the corresponding author on reasonable request.

## Abbreviations

*c*: Specific heat capacity (J kg^−^^1^ °C^−^^1^)
*d*: Test duration (h)
ESE: East–southeast
GSHP: Ground source heat pump
GSM: Global system for mobile communication
m′: Mass flow rate (kg s^−^^1^)
*P*_max_: Peak thermal power (W)
*P*_mean_: Mean thermal power (W)
PE-D: High-density polyethylene
Pe-Xa: Reticulated polyethylene
*Q*_mean_: Mean exchange rate (W m^−^^2^)
*q*: Volumetric flow rate (l/h)
SGE: Shallow geothermal energy
*T*: Temperature (°C)
*T*_a_: External air temperature (°C)
*T*_i_: Air temperature in the cavaedium (°C)
*T*_lin_: Temperature of the fluid in the line pipe (°C)
*T*_ret_: Temperature of the fluid in the return pipe (°C)
*T*_T_: Target temperature of the test at the user side (°C)
*λ*: Thermal conductivity (W m^−^^1^ °C^−^^1^)
*ρ*: Specific weight (kg m^−^^3^)
